# Integration of ATAC-seq and RNA-seq reveals temperature-responsive regulatory regions in *Plasmodium falciparum* asexual stages

**DOI:** 10.1186/s13071-026-07288-2

**Published:** 2026-02-20

**Authors:** Yuhong Zhang, Yi Zhao, Guixing Zheng, Shanli He, Jiale Xiao, Haochen Ma, Jun Huang, Yanwei Qi

**Affiliations:** 1 Department of Pathogenic Biology and Immunology, Sino-French Hoffmann Institute, School of Basic Medical Sciences, Guangzhou Medical University, Guangzhou, 511436 Guangdong China; 2 Scientific Research Center, Guangzhou Medical University, Guangzhou, 511436 Guangdong China; 3 Department of Blood Transfusion, The First Affiliated Hospital, Guangzhou Medical University, Guangzhou, 510120 Guangdong China; 4 The Second School of Clinical Medicine, Guangzhou Medical University, Guangzhou, 511436 Guangdong China

**Keywords:** Chromatin accessibility, *Plasmodium falciparum*, ATAC-seq, RNA-seq, Temperature-responsive

## Abstract

**Background:**

Malaria remains a critical parasitic disease in tropical regions, with environmental temperature significantly influencing the development and transmission of *Plasmodium falciparum*. While low temperature triggers gametocyte differentiation in mosquito, the molecular mechanisms underlying temperature-responsive chromatin and transcriptional dynamics in asexual stages (ring and trophozoite) remain unclear. This study integrates Assay for Transposase-Accessible Chromatin with Sequencing (ATAC-seq) and RNA-sequencing (RNA-seq) to characterize genome-wide chromatin accessibility and gene expression profiles in *P. falciparum* under human body temperature (37 °C) and mosquito-mimicking temperature (26 °C).

**Methods:**

Synchronized ring (45 h post-invasion) and trophozoite (70 h post-invasion) stages were subjected to temperature treatments (37 °C versus 26 °C). ATAC-seq was used to identify accessible chromatin regions, RNA-seq analyzed differentially expressed genes (DEGs), and quantitative real-time polymerase chain reaction (qPCR) validated key gene expression changes.

**Results:**

Low temperatures exerts a profound impact on the activation and expression of sexual-stage-specific genes in *P. falciparum* and induced 1083 differentially accessible regions (DARs) in the ring stage, including 1081 gains and only 2 losses, which were enriched primarily in promoter regions (≤ 3 kb upstream of transcription start sites), whereas no significant DARs were detected in the trophozoite stage, indicating stage-specific sensitivity to temperature. Functional analyses revealed DAR-associated genes enriched in host cell membrane interactions, antigenic variation, and pathways such as symbiont-mediated perturbation of host erythrocyte aggregation, with the identification of temperature-responsive transcription factor motifs (e.g., DEAR-3, ERF096). Integration of ATAC-seq and RNA-seq revealed a positive correlation between chromatin accessibility and gene expression, with 41 genes exhibiting concordant DAR-DEG changes, suggesting that dynamic chromatin remodeling regulates temperature-responsive transcription.

**Conclusions:**

Low temperature selectively modulates chromatin accessibility and gene expression in the ring stage, while trophozoites show no chromatin reconfiguration, highlighting stage-specific thermal sensitivity. This study represents the first integrative analysis of ATAC-seq and RNA-seq data from *P. falciparum* under low temperature stress, identifying critical temperature-responsive regulatory regions, providing insights into the parasite’s environmental adaptation and a foundational resource of temperature-responsive regulatory regions, whose future functional validation could inform the development of novel, chromatin-targeted antimalarial strategies.

**Graphical Abstract:**

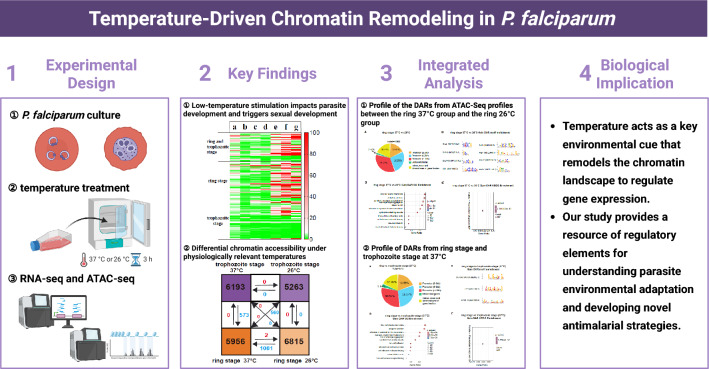

**Supplementary Information:**

The online version contains supplementary material available at 10.1186/s13071-026-07288-2.

## Background

Malaria persists as one of the most formidable parasitic diseases in tropical regions, disproportionately affecting numerous developing countries and exacting a heavy toll in terms of morbidity and mortality. According to the World Health Organization (2024), approximately 240 million malaria cases and 600,000 malaria-related deaths are reported annually in endemic countries as of 2023, predominantly in the sub-Saharan African region [1]. Despite the implementation of diverse control strategies, including vector management, bed net utilization, and chemotherapy, the outcomes have been only moderately successful [2]. The absence of effective vaccines, the widespread emergence of resistance to existing antimalarial drugs [3], and the incomplete understanding of underlying molecular mechanisms underscore the urgent need for innovative and efficacious malaria control approaches [4]. Temperature fluctuations exert a nonuniform and disruptive influence on various cellular processes [5]. For *Plasmodium*, precise temperature regulation is critical for its development, as environmental temperature directly modulates the pace and progression of *P. falciparum* infection within both human hosts and mosquitoes [6, 7]. The differentiation of gametocytes within mosquitoes is a pivotal step in the parasite transmission cycle, and a temperature decrease of approximately 5 °C serves as a key trigger for malaria gametogenesis. However, the molecular mechanisms governing gametocyte differentiation remain largely elusive.

In eukaryotic organisms, the genome is intricately organized at the chromatin level. Regulatory elements, such as promoters and enhancers, serve as access points for controlling gene activity [8]. In *Plasmodium*, these accessible regions constitute about 1% of the genome, identifiable by techniques such as formaldehyde-assisted isolation of regulatory elements (FAIRE) and Assay for Transposase-Accessible Chromatin with high throughput sequencing (ATAC-seq) [9, 10]. While studies in mammalian systems show these elements interact with transcription factors to govern cell fate [11, 12], the mechanisms of chromatin reconfiguration during the *Plasmodium* developmental cycle remain unclear. Since its development by Buenrostro et al. in 2013, ATAC-seq has emerged as a powerful, genome-wide technique that enables comprehensive analysis of chromatin accessibility [13]. This method has been applied across a wide range of species, including mouse cell lines [14], *Epinephelus coioides* [15], *Schizochytrium limacinum* [16], *Arabidopsis thaliana* [17], *Caenorhabditis elegans* [18], zebrafish [19], livestock [20], parasite vector mosquitoes [21], and different cell types in the human body [22]. Investigating the accessible chromatin structure associated with biological events is essential for deciphering the functional roles of these chromatin elements.

Dynamic chromatin elements, which can switch between accessible and inaccessible states, represent an additional layer of the genetic code that is still in the early stages of characterization [23]. Understanding these complex global chromatin structures is fundamental to elucidating the mechanisms underlying RNA transcript function and regulation [24]. Chromatin accessibility is highly responsive to changes in cellular conditions and stimuli, including temperature [25]. Temperature is a key regulator of *Plasmodium* sexual stage development [26, 27]. The parasite is highly sensitive to temperature fluctuations, with the human host’s body temperature (37 °C) triggering significant metabolic changes. Conversely, the optimal temperature for development in mosquitoes is 26 °C. Exposure to this lower temperature can initiate sexual stage development, activating gametocytes and transmission-related genes [28]. The transmission of *Plasmodium* parasites to mosquitoes is contingent upon the differentiation of intra-erythrocytic forms into sexual gametocytes within the human host, and the parasite maintains a delicate balance between transmission and persistent infection through as-yet-undefined mechanisms [29]. Although the asexual stages of the parasite do not naturally encounter 26 °C within the human host, this temperature mimics the mosquito transmission environment, providing a unique opportunity to study the adaptive chromatin responses of the parasite to thermal stress. Previous studies have utilized temperature shifts from 37 °C to 26 °C to investigate the effects on *P. falciparum* development and gene expression [6, 29–31]. These studies have shown that such temperature changes can have a significant impact on the development and gene expression of the parasites. Although asexual stage parasites do not directly experience these temperature changes in the natural host vector cycle in the same way, this experimental system allows us to mimic the environmental stressors that the parasite may encounter and study its response at the molecular level. Our preliminary data also indicate that the observed changes in chromatin accessibility and gene expression are consistent with the parasite’s adaptation to different thermal environments.

The ring and trophozoite stages of *Plasmodium* are central to the asexual blood cycle, during which the parasite replicates and causes disease in the host. These stages are more abundant and experimentally tractable compared with gametocytes, which are crucial for transmission but are less abundant and more challenging to culture. Analyzing the asexual stages can provide valuable insights into conserved regulatory mechanisms that operate across different lifecycle stages. Our analysis revealed at least 3340 and 4688 overlapping peaks (also known as accessible chromatin regions) in the ring and trophozoite stages, respectively, at 37 °C, and 4695 and 4467 overlapping peaks at 26 °C. Our results provide a basis for understanding how the parasite adapts to its thermal environment through in vivo chromatin structural modifications, laying the groundwork for future research. Additionally, our findings suggest a crucial link between chromatin structure and the intricate biological processes of *P. falciparum* following exposure to low temperature stimuli.

The major strength of this study lies in its demonstration of how temperature influences parasite development through the modulation of chromatin structure. To our knowledge, this study represents the first comprehensive integration of ATAC-seq and RNA-seq datasets to investigate *P. falciparum* under conditions of low temperature stress. By comparing these datasets, we have identified key cis-regulatory elements associated with low temperature stress-specific genes. These findings not only advance our understanding of host–parasite interactions and parasites’ environmental adaptation, but also provide valuable resources for future studies aimed at identifying gene regulatory elements involved in parasite developmental transitions. Ultimately, these insights provide a foundation for the development of chromatin-targeted antimalarial strategies, which may offer new avenues for therapeutic intervention.

## Results

### Low-temperature stimulation impacts parasite development and triggers sexual development

**Fig. 1:**
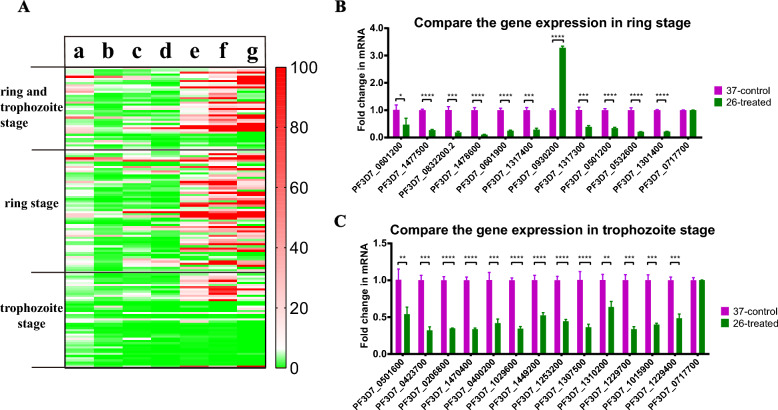
Comparative analysis of temperature-dependent gene expression in *Plasmodium*: insights from RNA-seq and qPCR studies across multiple stages. **A** Overlap of genes upregulated in ring and trophozoite stages at 26 °C from this study with a previous RNA-seq dataset across seven *P. falciparum* stages (a–g: ring to ookinete). **B**, **C** qPCR validation of gene expression changes (26 °C versus 37 °C) in ring (**B**) and trophozoite (**C**) stages. At the bottom, name of each gene is listed. These results represent data from three independent experiments. Statistical analysis was performed via a two-sided *t* test, where significance levels are denoted as *P < 0.05, **P < 0.01, ***P < 0.001, ****P < 0.0001, or not significant (ns)

Previously, we subjected the parasites to temperature stimuli (37 °C and 26 °C) at 45/70 (ring stage/trophozoite stage) h after initial synchronization and cultured for 3 h to determine the effect of low temperature on the dynamics of parasite development and gene expression [31]. The reproducibility of our RNA-seq data has been validated in a previous study [31], with a Pearson’s correlation coefficient of ≥ 0.937 between biological replicates, confirming the reliability of the differential expression patterns reported herein. The RNA-seq results indicated that several genes were differentially expressed among the different groups in response to low temperature stimuli (164 genes for rings and 187 genes for trophozoites are differentially expressed at 26 °C versus 37 °C) and parasites at different stages and temperatures exhibit varying gene expression profiles. To investigate whether cold temperature induction triggers sexual development in *P. falciparum*, we statistically analyzed genes showing upregulated expression at two developmental stages under low temperature stimulation (Fig. 1A). Specifically, we compared these genes with expression profiles across seven developmental stages (ring, early trophozoite, late trophozoite, schizont, gametocyte II, gametocyte V, and ookinete) reported in previous study [32].

RNA-seq results demonstrated that genes upregulated in ring and trophozoite stages following low temperature stimulation (26 °C) predominantly exhibited high expression in sexual stages (gametocyte II, gametocyte V, and ookinete) in the published dataset. This finding indicates that cold temperature exposure significantly influences the activation and expression of sexual stage specific genes in *P. falciparum*. Notably, even though the experimental system utilized asexual erythrocytic stages (ring and trophozoite), which are typically unrelated to gametocyte or ookinete stages in mosquitoes, exposure to low temperature (26 °C) was sufficient to induce developmental programs analogous to those in the mosquito vector. Low temperature can recapitulate molecular events underlying gametocyte differentiation in mosquitoes, providing a valid experimental model to study early sexual development in *P. falciparum*. These results imply that temperature could be a crucial regulatory factor linking parasite development with malaria transmission. In previous studies, we compared the structural changes in RNA under normal culture temperature conditions and low temperature induction and reported that low temperature influences parasite development at the RNA secondary structure level [31]; however, its impact on parasite chromatin structure dynamics remains to be elucidated. Extensive chromatin structure and parasite developmental diversity hinder functional analysis of all linked genes or motifs.

### Validation of the Results via qPCR

To validate the accuracy of RNA-seq in assessing the expression pattern alterations induced by low temperature stimuli, quantitative real-time PCR (qPCR) was used to measure the fold changes in the expression levels of some genes. We randomly selected the 25 genes for qPCR validation on the basis of their biological relevance (e.g., Glycosylphosphatidylinositol (GPI)-anchor biosynthesis, antigenic variation) and statistical significance in our experimental conditions. The 25 differentially expressed genes (DEG) confirmed by qPCR represent a subset of the total DEG identified in our RNA-seq analysis. A comparison of the altered transcripts during ring stage development at 37 °C and 26 °C revealed 12 genes distributed across various parasite chromosomes: 1 gene was upregulated, 10 genes were downregulated, and the remaining gene was a housekeeping gene, serine-tRNA ligase (PF3D7_0717700), which served to normalize the transcriptional levels (Fig. 1B). Furthermore, in the trophozoite stage of development, 14 genes situated on various parasite chromosomes were identified: 13 genes exhibited downregulation, with the remaining gene being the housekeeping gene (Fig. 1C). All 25 putative DEGs identified via RNA-seq were confirmed via qPCR and those genes showed concordant expression changes, supporting the reliability of RNA-seq data. For qPCR validation, three biological replicates were performed for each sample, with three technical replicates for each biological sample to ensure consistency and reliability. Information on the primers and oligonucleotide sequences utilized in the experiment is provided in Table S1. The list of genes whose mRNA levels were detected via qRT‒PCR is shown in Fig. 1. These findings suggest the reliability and reproducibility of the transcriptome data and gene expression profiles identified via RNA-seq, supporting their potential as candidate genes for further investigation into low-temperature-induced gametocyte differentiation or developmental stages.

### Assessment of the quality of ATAC-seq data

**Fig.2:**
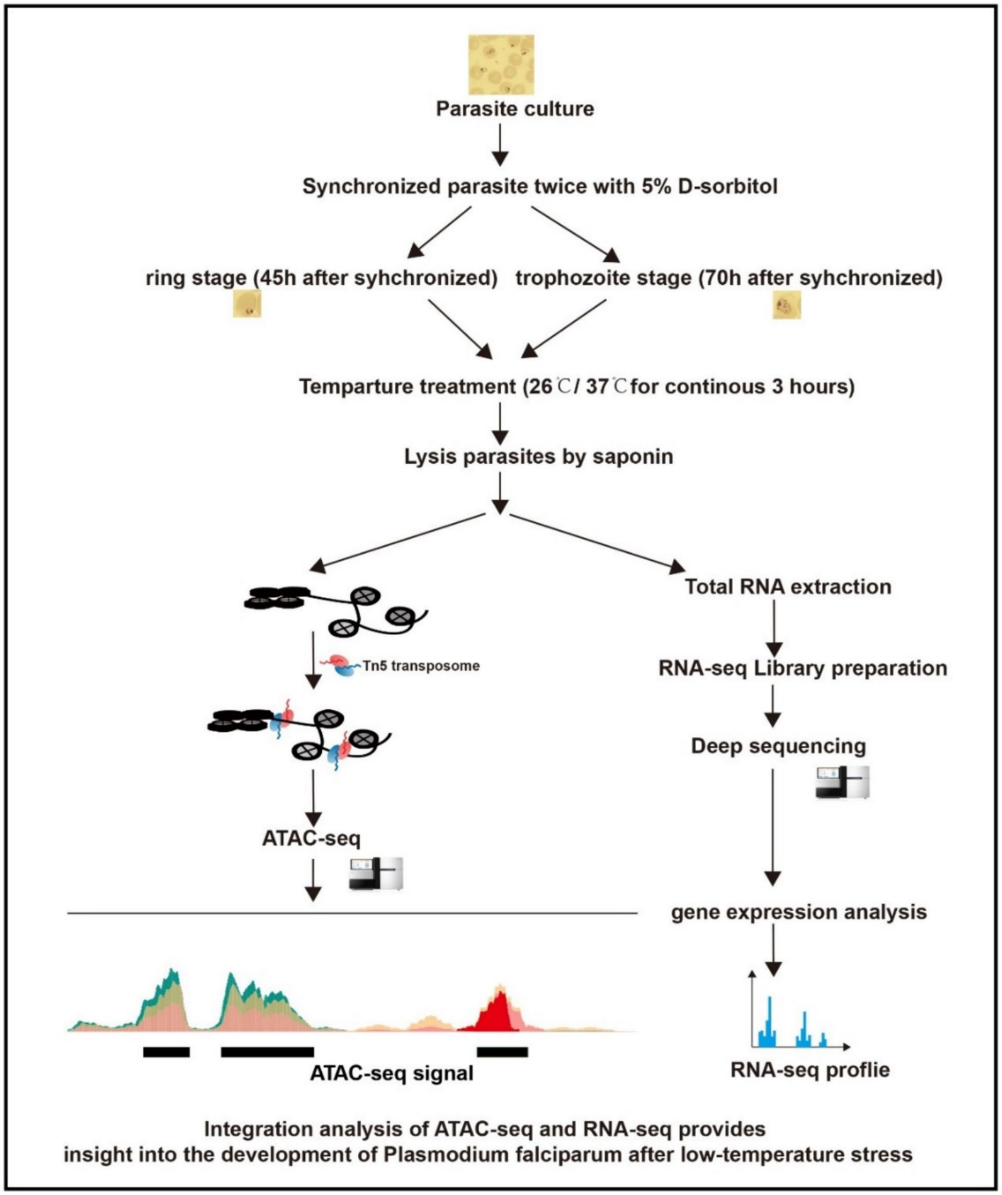
Experimental workflow for ATAC-seq and RNA-seq analysis. Synchronized *P. falciparum* cultures at ring (45 h post-invasion) and trophozoite (70 h post-invasion) stages were subjected to 37 °C or 26 °C for 3 h. Parasites were then processed for parallel ATAC-seq and RNA-seq library preparation from the same culture

For a comprehensive genome-wide characterization of open chromatin regions throughout the intraerythrocytic developmental cycle of *P. falciparum*, experiments were conducted at both 26 °C (vector host body temperature) and 37 °C (human host body temperature), and we constructed an ATAC-seq library from the same parasite cultures used for RNA-seq, as illustrated in Fig. 2. Temperature stimuli (37 °C and 26 °C) were applied 45 h and 70 h after the initial synchronization, corresponding to the ring and trophozoite stages, respectively. The parasites were then maintained at these temperatures for 3 h, following previously established protocols.

**Fig. 3:**
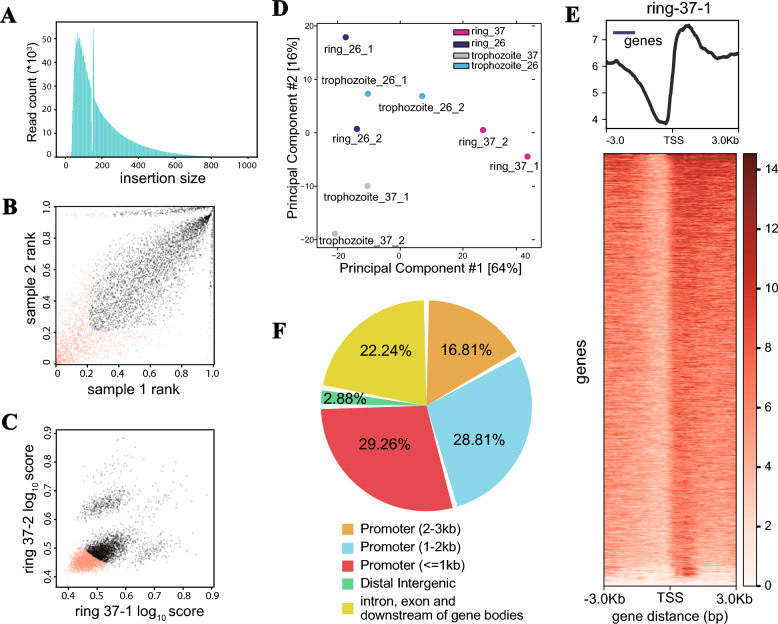
Assessment of the quality of ATAC-seq data. **A** Fragment lengths within a representative ATAC-seq library. The majority of the fragment lengths were small fragments (< 200 bp, internucleosomal open chromatin), and no fragment enrichment occurred at approximately 146 bp. The robustness of the ATAC-seq raw data was evaluated by evaluating the data repeatability in a single sample (**B**) and parallel samples (**C**), which were highly correlated. **D** Principal component analysis (PCA) of ATAC-seq data. Each point represents a sample, with colors indicating developmental stages and shapes indicating temperature conditions. Samples from the same group cluster closely, demonstrating strong biological reproducibility and distinct group separation. **E** Distribution of ATAC-seq peak regions across different genome annotations. All the data presented in this figure pertain to sample ring stage 37-1. **F** ATAC-seq signals were concentrated around the transcriptional start sites (TSSs) in a typical sample. The top panel displays the combined enrichment plot encompassing all the TSSs. The heatmap illustrates the enrichment surrounding a specific TSS. *TSS* transcriptional start site

For ATAC-seq, we followed the procedures outlined by Corces et al. (2017) as described previously [13, 33], with some modifications. After the raw data were obtained from ATAC-seq, the fragment size of each read pair from the Binary Alignment/Map (BAM )file of paired-end sequencing was calculated. Peak calling statistics for all samples are provided in Table S2. In our ATAC-seq analysis, we achieved an average mappability rate of 53.96% and obtained approximately 12.1 million qualified fragments per sample (Table 1). First, all ATAC-seq libraries presented the expected fragment lengths with the expected distribution, an important index for evaluating ATAC-seq data quality, including the majority of small fragments (< 200 bp, internucleosomal open chromatin), an obvious fragment enrichment, which is a completely nucleosome-free region (NFR) at 40–60 bp, and no fragment enrichment, at approximately 146 bp, because of the occupying effect of nucleosomes, indicating good data quality (Fig. 3A). This finding suggested that many fragments identified via ATAC-seq were protected by integer multiples of nucleosomes. Notably, similar fragment distributions have been reported in previous ATAC-seq studies of *P. falciparum* [13, 15, 34], supporting the reliability of our data.

**Table 1:** Summary of the ATAC-seq data

Sample	Raw reads	Clean reads (%)	Aligned reads	Align rate (%)	Total peak
ring_37_1	27,075,556	27,018,398 (99.79)	10,839,026	0.40	4200
ring_37_2	23,814,314	23,802,876 (99.95)	20,608,370	0.87	8489
ring_26_1	27,722,114	27,714,512 (99.97)	20,070,712	0.72	8370
ring_26_2	14,063,944	14,059,922 (99.97)	10,085,643	0.72	5262
trophozoite_37_1	65,333,314	65,066,424 (99.59)	9,785,745	0.15	6030
trophozoite_37_2	19,518,772	19,506,966 (99.94)	11,081,055	0.57	6335
trophozoite_26_1	16,495,318	16,492,138 (99.98)	11,393,369	0.69	6449
trophozoite_26_2	22,658,354	22,619,478 (99.83)	2,959,625	0.13	4699

Second, the robustness of the ATAC-seq raw data was evaluated by evaluating the data repeatability in a single sample (Fig. 3B) and parallel samples (Fig. 3C), which were highly correlated. Furthermore, we employed a stringent iterative discovery rate (IDR) method to assess the consistency of the peak enrichment ratios. This statistical approach operates on replicated peak sets, comparing the ranks of peaks within individual replicate or pseudoreplicate sets. Our findings indicate clear clustering of samples by group, with closely aligned samples within each group, confirming high-quality ATAC-seq results. To further evaluate sample clustering and biological reproducibility, we performed principal component analysis (PCA) on the ATAC-seq dataset. As shown in Fig. 3D, samples from the same developmental stage and temperature condition cluster tightly together, indicating minimal technical variation and distinct biological signatures between groups. Additionally, peak overlap analysis between biological replicates revealed higher consistency across all experimental groups (Supplementary Fig. S1A), confirming robust reproducibility of the accessible chromatin regions identified. These results collectively validate the high quality and reliability of our ATAC-seq data.

Third, we asked whether it is usually enriched in the vicinity of the transcription start site (TSS), the specific binding sites that bind transcription factors to gene promoters among all the putative accessible genomic regions detected by ATAC-seq. As shown in Fig. 3E, there was strong enrichment around the TSS (highly open regions of the genome) and an increase in the signal up to a peak in the middle. Additionally, no less than 75% of the putative accessible genomic regions were located 3 kb upstream of the TSS, especially (average 33.3% in all eight samples) the promoter 1 kb upstream of the TSS; approximately 20.4% were located in introns, exons, and downstream regions of gene bodies; and the remaining 1.82% were located in distal intergenic regions (Table S3 and Fig. 3F). The results of the mapped read distribution on the gene body and gene peak show that the ATAC sequence has good quality and can reproducibly and reliably determine chromatin accessibility in these samples. Therefore, despite the AT-rich genome of *P. falciparum*, our ATAC-seq method is still capable of robust and accurate identification of accessible chromatin regions.

For each individual sample, valid high-confidence peaks were identified, with gene coverage metrics as follows (Table S3): in ring-stage samples, ring_37_1 had 4200 peaks covering 2750 genes (47.49% of all genes); ring_37_2 8489 peaks (3811 genes, 65.81%); ring_26_1 8370 peaks (4207 genes, 72.65%); and ring_26_2 5262 peaks (3437 genes, 59.35%). In trophozoite-stage samples, trophozoite_37_1 yielded 6030 peaks (3524 genes, 60.85%); trophozoite_37_2 6335 peaks (3646 genes, 62.96%); trophozoite_26_1 6449 peaks (3728 genes, 64.38%); and trophozoite_26_2 4699 peaks (3004 genes, 51.87%). These data complement the merged replicate results, providing detailed peak characteristics for each individual sample and further confirming the robustness of ATAC-seq data in capturing accessible chromatin regions across different experimental conditions (Table S3). Notably, the proportion of peaks in the promoter (2–3 kb) region, ranging from 14.71% to 17.66% across all samples, serves as direct evidence of our assay’s ability to effectively capture distal regulatory sites beyond the immediate proximal promoter. The sequencing depth is sufficient to recover most of the chromatin accessible regions in each sample. Since their initial identification, the ApiAP2 family, recognized as the primary group of putative transcription factors in *Plasmodium*, has been demonstrated to control a nuanced gene expression program through individual or combined actions [35–37]. With all 28 putative ApiAP2 TFs, 28 ApiAP2 family genes were detected via ATAC-seq analysis (Table S4), suggesting potential involvement in temperature response.

To assess the consistency of our ATAC-seq data with previous studies, we compared our peaks with those from Ruiz et al. (2018) and Toenhake et al. (2018), which focused on overlapping stages (ring and trophozoite at 37 °C). Our data overlapped with 33.8–74.4% of peaks from each reference, with higher overlap in trophozoite stages (data not shown). Our biological replicates showed > 80% overlap (Fig. S1A), confirming robust reproducibility. RUF6-associated accessible sites [38] overlapping with our peaks correlated with *var* gene expression dynamics (Fig. S1B). As shown in Fig. S1C, motif enrichment analysis in our research revealed significant overlap with published motifs [39], including the AP2-I binding site GTGCA, underscoring the conservation of regulatory. These results indicate that our dataset captures functionally relevant accessible regions while identifying novel temperature-responsive loci.

### Differential chromatin accessibility under physiologically relevant temperatures

**Fig. 4:**
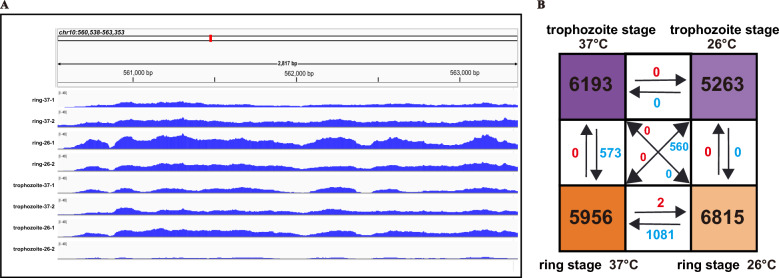
Differential chromatin accessibility under temperature stress. **A** Browser tracks of ATAC-seq signals at the PF3D7_1014200 locus (chromosome 10) for all eight samples. **B** Number of DARs observed among groups under various temperatures and developmental conditions. The numbers annotated in different colored backgrounds represent the number of peaks identified under each respective treatment condition

ATAC-seq analysis revealed 6692 unique and common peaks in *P. falciparum* at two developmental stages and under two distinct temperature conditions (Table S5). This result was obtained after preprocessing eight MACS-called peak sets (deduplication and merging) and calculating read counts for each peak region using corresponding BAM files, with DiffBind-processed peaks showing a consistent width of 401 bp (deviating from the original MACS-called peaks). To assess whether the accessible chromatin was successfully detected and the reproducibility of replication, ATAC-seq peaks associated with several temperature-related genes were strongly enriched. PF3D7_1014200, male gamete fusion factor HAP2 [40, 41], which plays an essential role in microgamete fertility and mediates gamete fusion during fertilization, was significantly enriched in the mosquito transmission environment (26 °C) group but was less/not detected in the host body (37 °C) temperature groups in the ring stage (Fig. 4A).

These data also revealed some specific or shared peaks among different groups. For example, some peaks associated with 20 genes of the *var* gene family (Table S6) are shared by the two different groups and temperatures when the average threshold of the peak is set to 5. In the ring stage, one peak associated with eukaryotic translation initiation factor 3 subunit G, putative (EIF3G, PF3D7_0815600) was specific to the 37 °C group, whereas another peak associated with the asexual protein 2 (CLAG2, PF3D7_0220800) gene was specific to the 26 °C group (Table S7). Additionally, in the trophozoite stage, one peak associated with the transcription factor with AP2 domain(s) (ApiAP2, PF3D7_1350900) is group specific at 37 °C, and another peak with serine/threonine protein kinase RIO2 (RIO2, PF3D7_0420100) gene is group specific at 26 °C. Overall, at least 1811 genes presented no less than one ATAC-Seq peak in each group; 333 peaks were ring 37 °C specific, 145 peaks were ring 26 °C specific, 45 peaks were trophozoite 37 °C specific, and 20 peaks were trophozoite 26 °C specific (Table S7).

We initially compared DARs during the trophozoite stage under two temperature conditions. Surprisingly, we found that the lower temperature stimulus at 26 °C did not affect the chromatin accessibility of the parasite during this stage, revealing no DAR. These findings indicate that low temperature does not influence chromatin accessibility of the trophozoite stage. However, we identified 1081 gain and 2 loss of DARs in the 26 °C ring group relative to the 37 °C ring group (Fig. 4B). A comparison of the differences observed under low temperature stimulation between the ring stage and the trophozoite stage revealed no variation between the two stages. Specifically, the trophozoites presented no DAR compared with the ring stage. These results suggest that under 26 °C conditions, there is little disparity in chromatin accessibility between these two stages, indicating nearly equivalent levels of chromatin accessibility. We also compared DARs among different temperatures and developmental stages of malaria parasites. When the ring stage at 37 °C was compared with the late trophozoite stage at 26 °C, there were significant differences, with 560 gain DARs and no loss DARs, as shown in Fig. 4B. Conversely, when the late trophozoite stage at 37 °C was compared with the ring stage at 26 °C, there were no significant DARs. To some extent, the ring stage after 26 °C low temperature stimulation exhibited chromatin accessibility patterns that resembled those of trophozoite stage under both 37 °C and 26 °C conditions.

### Differential chromatin accessibility during low temperature responsive in the ring stage

**Fig. 5:**
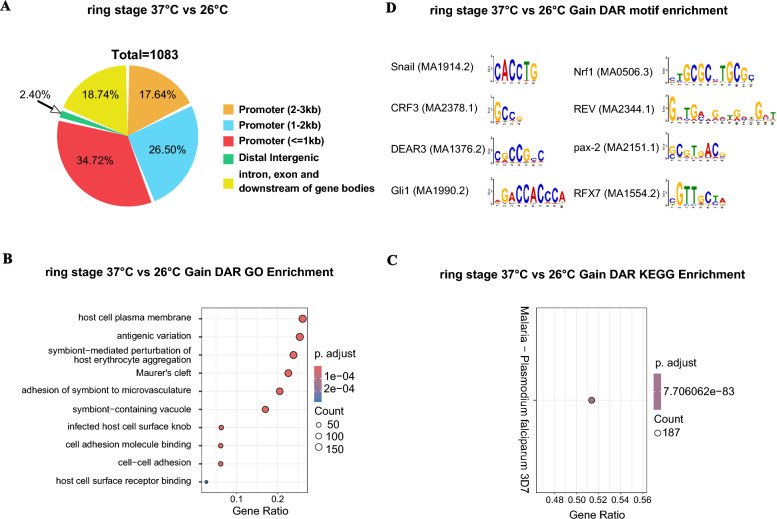
Profile of the DARs from ATAC-Seq profiles between the ring 37 °C group and the ring 26 °C group. **A** Proportions of DARs that represent the various genome annotations in the ring stage. The DARs of the ATAC-Seq profiles among all the samples were calculated via DiffBind software with a filtering threshold of log_2_ fold change >  = 0.5 and *P*-value <  = 0.05. **B** GO annotation of these gain and loss DARs in the 26 °C ring group relative to the 37 °C ring group; **C** KEGG enrichment analysis of these gain and loss DARs in the 26 °C ring group relative to the 37 °C ring group; related to Table S9. **D** Motif analysis of these gain DARs in the 26 °C ring group relative to the 37 °C ring group; related to Table S9

To identify and characterize TF-binding events in parasites, we analyzed the DARs across all four groups via DiffBind software. We applied a filtering threshold of |log_2_ fold change|> = 0.5 and a significance threshold of *P* value <  = 0.05. We focused on the significant DARs observed between ring stages. There were 1081 gain and 2 loss DARs in the 26 °C ring group relative to the 37 °C ring group (Fig. 5A and B and Table S8). A total of 78.86% of the putative DARs were located 3 kb upstream of the TSS, especially the promoter 1 kb upstream of the TSS (34.72%); approximately 18.74% were located in introns, exons, and downstream regions of gene bodies; and the remaining 2.40% were located in distal intergenic regions (Fig. 5A; Table 2).

**Table 2:** Numbers and proportions of DAR in ring 26 °C group, relative to ring 37 °C group

	All DAR	Gain DAR	Loss DAR
Promoter (2–3 kb)	191	191	0
Promoter (1–2 kb)	287	287	0
Promoter (< = 1 kb)	376	374	2
Distal intergenic	26	26	0
Intron, exon, and downstream of gene bodies	203	203	0
Total	1083	1081	2
Promoter (2–3 kb) (%)	17.64	17.67	0.00
Promoter (1–2 kb) (%)	26.50	26.55	0.00
Promoter (< = 1 kb) (%)	34.72	34.60	100.00
Distal intergenic (%)	2.40	2.41	0.00
Intron, exon, and downstream of gene bodies (%)	18.74	18.78	0.00

According to the Gene Ontology (GO) annotation of these DARs, the top three gene ontology enrichments in gain DARs were antigenic variation, host cell plasma membrane, and symbiont-mediated perturbation of host erythrocyte aggregation (Fig. 5B and Table S9). Kyoto Encyclopedia of Genes and Genomes (KEGG) enrichment analysis revealed that gain DARs were associated with malaria—*Plasmodium falciparum* 3D7 (Fig. 5C and Table S9). Due to the limited number of loss DARs (only two), neither Gene Ontology (GO) nor Kyoto Encyclopedia of Genes and Genomes (KEGG) enrichment analyses yielded significant results for this category. Finally, we discovered several specific motifs in the gain DARs (Snail, CRF3, DEAR3, Gli1, Nrf1, pax-2, REV, RFX7, etc.) related to temperature stimuli (Fig. 5D and Table S9).

### Differential chromatin accessibility at two developmental stages under the same temperature

**Fig. 6:**
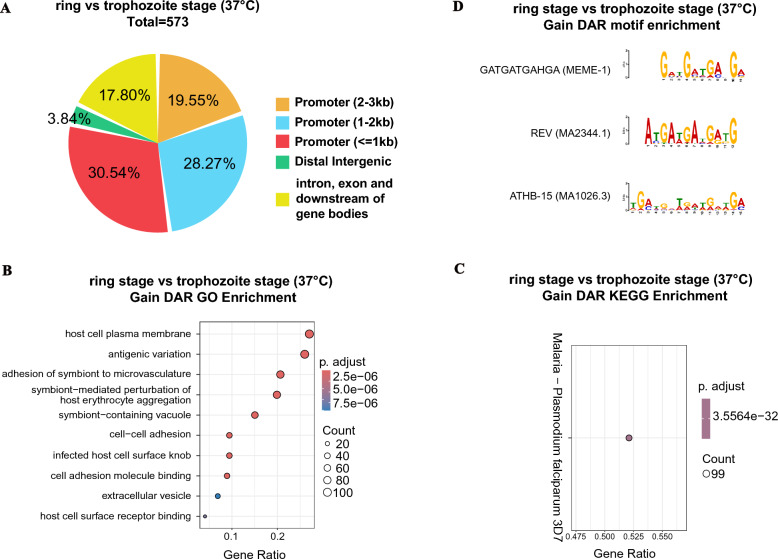
Profile of DARs from ring stage and trophozoite stage at 37 °C. **A** Proportions of DARs that represent the various genome annotations. The DARs of the ATAC-Seq profiles between two samples were calculated via DiffBind software with a filtering threshold of log_2_ fold change >  = 0.5 and a *P* value <  = 0.05. **B** Gene ontology annotation of these gain and loss DARs in the ring stage at 37 °C relative to the trophozoite stage at 37 °C; **C** KEGG enrichment analysis of these gain DARs in the ring stage at 37 °C relative to the trophozoite stage at 37 °C; related to Table S11. **D** Motif analysis of these gain DARs in the ring stage at 37 °C relative to the trophozoite stage at 37 °C; related to Table S11

We identified local regions of different accessibility from two stages at a human host body temperature of 37 °C via DiffBind software with the same filtering method, and the results revealed that a huge number of genes had DARs in different groups. Compared with the ring stage parasites in the 37 °C control group, there are 573 gain DARs in the trophozoite stage group, as shown in Fig. 6A and Table S10. These DARs included erythrocyte membrane protein 1 (PfEMP1, VAR, PF3D7_0324900, PF3D7_0420700, PF3D7_0400200), sentrin-specific protease 2 (putative, SENP2, PF3D7_0801700), and some conserved *Plasmodium* proteins with unknown functions. As shown in Fig. 6A, those stage-specific DARs also had high consistency in parallel groups. A total of 78.36% of the putative DARs were located 3 kb upstream of the TSS, especially the promoter 1 kb upstream of the TSS (30.54%) (Fig. 6A).

The detailed GO and KEGG enrichment results for the 573 DARs associated with developmental switching are shown in Fig. 6B and C and Table S11. The top three GO enrichment in gain DARs were the host cell plasma membrane, antigenic variation, and adhesion of symbiont to microvasculature (Fig. 6B). The KEGG enrichment analysis revealed that these gain DARs were associated with malaria—*Plasmodium falciparum* 3D7 (Fig. 6C). These KEGG enrichment results were similar to those related to different developmental stages. We also discovered some specific motifs in gain DARs (GATGATGAHGA (MEME-1), REV (MA2344.1), ATHB-15 (MA1026.3), etc.) related to differential developmental stages. These specific motifs can provide guidance for future studies (Fig. 6D).

### Integration of ATAC-seq and RNA-seq reveals key genes involved in low-temperature-induced parasite differentiation

**Fig. 7:**
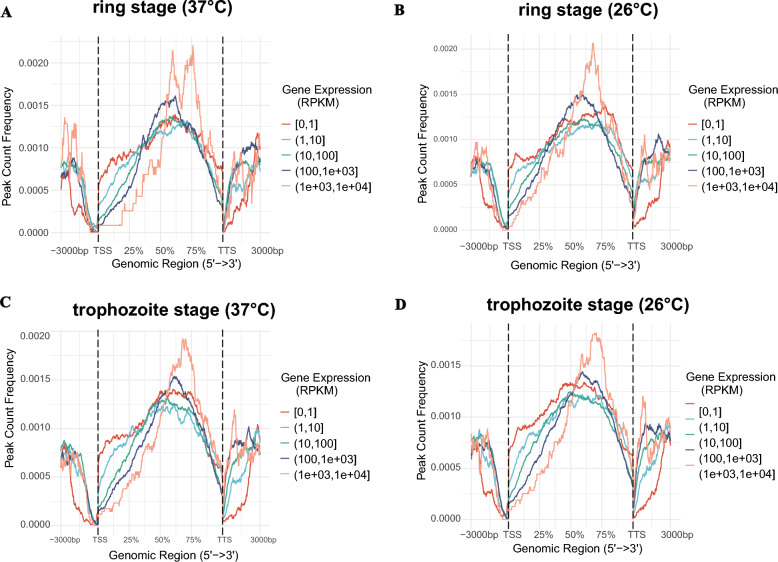
Distribution of ATAC-seq signals across the gene body. **A** Correlations between ATAC-Seq signal intensity and gene expression levels during the ring stage at 37 °C. **B** Associations between ATAC-Seq signal intensity and gene expression levels during the ring stage at 26 °C. **C** Connections between ATAC-Seq signal intensity and gene expression levels during the trophozoite stage at 37 °C. **D** Links between ATAC-Seq signal intensity and gene expression levels during the trophozoite stage at 26 °C

The binding of transcription factors to DNA sequences usually activates the expression of genes (including some repressive transcription factors); therefore, there is a corresponding relationship between the number of open chromatin regions and the expression level. Figure 7 shows the distribution of ATAC-seq signals across the gene body, as well as the 3 kb regions upstream and downstream, categorized into five levels on the basis of gene expression levels in each group. Our observations revealed a chromatin structure that was notably open, which was correlated with increased levels of transcriptional activity. These findings underscore that genes associated with highly accessible chromatin regions also presented elevated expression levels.

To investigate and more accurately determine the relationships and potential mechanisms of accessible chromatin regions from ATAC-seq data and expression profile changes caused by low temperature stimuli from RNA-seq, we integrated the results from ATAC-seq and RNA-seq, which were performed on the same cultured group samples. After overlapping, 20 upregulated genes with gain DAR in the promoter region were found in the ring stage at 37 °C and 26 °C, and 26 downregulated genes with gain DAR were found in the same group (Table S12). Only 20 genes showed concordant DAR–DEG changes, consistent with chromatin accessibility being one of multiple regulatory layers (e.g., post-transcriptional controls). Motif analysis suggests combinatorial TF regulation (Figs. 5D, 6D) may explain partial overlap.

The functional roles of these DARs in response to temperature or developmental stage remain unclear and warrant additional investigation. When we overlapped the two sequences resulting from both stages (37 °C), 21 upregulated genes with gain DAR 101 downregulated genes with gain DAR were found in the same group (Table S12). Moreover, our analysis revealed that several genes, including PF3D7_0413300, PF3D7_0801900, PF3D7_0831800, and PF3D7_1478600, contain multiple DARs distributed across distinct genomic regions (Table S12). These DARs encompass downregulated expression levels in two comparison, indicating a potential interplay between these opposing alterations. It is plausible that the combined effects of both types of DARs act synergistically to modulate the expression of these genes, thereby influencing their functional outcomes within the biological context. This finding suggests that not only activators, but also repressors, exist in *P. falciparum*. We observed that among the differentially expressed genes, some displayed increased DARs while others showed loss DARs. However, all of these changes in DARs were found to influence gene expression to varying extents.

## Discussion

### Rationale for experimental design and methodological validation

In this study, we used low temperature stimuli to induce parasite development and gametocyte differentiation in *P. falciparum*. The primary aim of this study was to investigate how the ring and trophozoite stages, despite not being directly exposed to the low temperature experienced by the mosquito vector, may still exhibit physiological responses to low temperature stimuli. We offer a comprehensive overview of the genome-wide landscape and dynamic chromatin structures of the significant human pathogen *P. falciparum* under two physiologically relevant temperatures, the human host body (37 °C) and the ambient environment (26 °C), mirroring mosquito transmission conditions. This is particularly relevant in the context of climate change and the potential expansion of vector-borne diseases. The ability of these stages to cope with environmental stimuli may enhance overall parasite fitness and survival in different ecological settings. Consequently, this adaptability is key to predicting how environmental changes might impact transmission dynamics. While focusing on asexual stages offers valuable information, there is a gap in understanding how temperature impacts gametocytogenesis, highlighting the need for future research. A key limitation of this study is that extrapolating results from in vitro 26 °C-treated asexual stages to in vivo transmission biology requires caution. The mosquito vector’s environment includes additional cues (e.g., pH changes, mosquito-derived factors) that synergize with temperature to regulate parasite development [42], and the observed chromatin and transcriptional changes in asexual stages likely represent pre-adaptive responses rather than direct mimicry of natural transmission events.

Environmental conditions, particularly temperature regulation, play crucial roles in the progression of *Plasmodium* infection. These environmental factors may alter epigenetic marks, such as heterochromatin-mediated gene silencing [43], which in turn can affect the rate and development of *P. falciparum*. However, how chromatin reconfigures and regulates transcription during temperature-controlled parasite development remains to be elucidated. An earlier study [44] on genome-wide profiling of chromosome interactions revealed that these interactions define the nuclear architecture of *P. falciparum*, with reconfigurations linked to antigenic variation. Two additional studies that performed ATAC-seq experiments on blood-stage *P. falciparum* parasites revealed temporal changes in accessibility and identified new transcription factor motifs, but the effects of chromatin structures on the development of parasites, especially after exposure to low temperature stimuli, remain controversial and are still in their infancy. Toenhake et al. (2018) conducted the first genome-wide ATAC-seq analysis and employed gDNA controls to correct for unspecific Tn5 tagmentation and intrinsic biases associated with the AT-rich genome of *P. falciparum* intraerythrocytic stages, identifying ~4000 regulatory regions predominantly located within 2 kb upstream of genes [10]. Their findings revealed a positive correlation between chromatin accessibility and mRNA abundance, with stage-specific regulatory regions driving reporter gene expression and containing motifs that interact with AP2 transcription factors. Building on this work, our study identifies temperature-responsive regulatory regions that are distinct from developmentally regulated elements, highlighting the parasite’s ability to modulate chromatin structure in response to environmental cues.

Our study extends this by demonstrating that low temperature selectively remodels chromatin in ring stages, with DARs enriched in promoter regions. Ruiz et al. (2018) characterized the accessible genome of *P. falciparum* across key life stages using ATAC-seq and integrated transcriptomics datasets from two different clones, revealing clonally variant gene expression patterns linked to chromatin accessibility [34]. Their work demonstrated that accessible regions are tightly associated with stage-specific gene expression and active histone modifications, providing a foundational resource for understanding transcriptional regulation in *P. falciparum*. Notably, while Ruiz et al. focused on clonal variation and developmental stage-specific chromatin regulation, our study extends this framework by investigating temperature-responsive chromatin dynamics in asexual stages, a previously unexplored dimension of environmental adaptation in *P. falciparum*. Our combined ATAC-seq/RNA-seq approach revealed 41 genes with concordant DAR-DEG changes, linking chromatin remodeling to transcriptional regulation under thermal stress. Through a transposase-accessible chromatin sequencing assay, we mapped chromatin-accessible regions in *Plasmodium*, analyzed transcriptome structural landscapes, and emphasized associations between distinct chromatin structures and their functions.

### Stage-specific chromatin remodeling in response to low temperature stress

An interesting observation of this study was that there was no DAR when we compared the two temperatures stimuli in the trophozoite stage, although there were some specific peaks. These results revealed an extensive difference in chromatin accessibility after exposure to temperature stimuli in the ring stage but not in the trophozoite stage, implying that DARs potentially represent highly stage-specific regulatory elements in *P. falciparum*. A striking finding of our study was the stage-specific nature of the chromatin response to low temperature stress, with significant changes in accessibility (DARs) predominantly occurring in ring stages but not in trophozoites. This differential susceptibility may be rooted in the distinct chromatin landscapes that characterize these developmental phases. Extensive epigenomic profiling has revealed that the *P. falciparum* genome undergoes profound reorganization throughout its intraerythrocytic cycle, with chromatin reaching a maximally “open” or accessible state during the trophozoite stage, which is associated with peak transcriptional activity [9, 45]. This established, stable chromatin architecture in trophozoites may be functionally “locked” into executing the essential transcriptional program for replication and schizogony, thereby rendering it less responsive to certain external stressors such as low temperature. In contrast to the “locked” chromatin architecture of the trophozoite stage, the ring stage exhibits greater chromatin plasticity.

These results allowed us to identify genes that exhibit different accessibilities in promoters at different temperatures, where switching between on/off states is associated with different gene expression patterns. According to GO annotation of these DARs, the top three GO terms were antigenic variation, host cell plasma membrane, and symbiont-mediated perturbation of host erythrocyte aggregation. KEGG enrichment analysis revealed associations of these DARs with malaria—*Plasmodium falciparum* 3D7 pathway. Moreover, we discovered several specific motifs (Snail, CRF3, DEAR3, Gli1, Nrf1, pax-2, REV, RFX7, etc.) related to temperature stimuli that can provide guidance for future studies. These may indicate conserved regulatory mechanisms, particularly given that 28 ApiAP2 transcription factor genes were detected in our ATAC-seq data, suggesting potential involvement of ApiAP2 family members in temperature response. The de novo motif analysis of temperature-responsive and stage-specific DARs identified several enriched sequence patterns. While initial annotation via the JASPAR database linked these to factors from model organisms (e.g., Snail, CRF3), we acknowledge the inherent limitation of such heterologous databases for *P. falciparum*, which possesses a unique repertoire of ApiAP2 transcription factors. Notably, while the AP2-I motif (GTGCA) is highly abundant in the global chromatin accessibility landscape, it was not enriched in the DARs, suggesting that the transcriptional response to temperature and developmental cues involves a more specialized regulatory program distinct from basal, stage-constitutive AP2-I activity. The identified motifs thus represent candidate cis-elements targeted by the transcription factor network that orchestrates the parasite’s adaptation to environmental stress and its developmental progression.

### Limited concordance between chromatin accessibility and transcriptional changes reveals multilayered regulation

In a prior investigation of *P. falciparum*, researchers reported temporal shifts in chromatin accessibility throughout development, revealing a robust correlation between these alterations and gene expression patterns [34]. In another previous study, chromatin accessibility analysis was employed to investigate potential epigenetic differences between the dormant forms on day 6 and the developing liver stage parasites. DiffBind was used to assess differential accessibility across all protein-coding gene promoters, identifying only 15 promoters with differential accessibility. Additionally, genes associated with heterochromatin features, which predict RNA binding, exhibited a significant reduction in accessibility. In contrast, genes associated with heterochromatic loss presented increased accessibility, although these differences did not reach statistical significance [46]. Our research revealed that chromatin structure undergoes dynamic changes during temperature-responsive development in *Plasmodium*. To accurately determine the relationships underlying these changes, we integrated ATAC-seq and RNA-seq data. After overlapping, some genes with gain/loss DAR in the promoter region with upregulated or downregulated expression were found in the groups at the ring stages of 37 °C and 26 °C. Therefore, our data clearly show that temperature-specific chromatin accessibility profiles are correlated with transcription changes. These findings suggest that activators and repressors exist in *P. falciparum* at the same time, which is consistent with the findings of previous studies [47–49]. A cascade of transcription-activating events, coupled with transcription-repressing events, are responsible for gene expression during blood stage development in response to low temperature stimuli. However, how the functional roles of DARs in the temperature response currently remain unclear and require further investigation. Notably, the coupling between the accessible chromatin region and the gene expression level in our analysis suggested that those genes are regulated by the nearest regulatory elements in parasites. Future high-resolution chromosome conformation studies, such as Hi-C sequencing, are necessary to explore distant enhancers that potentially correlate with specific transcriptional changes.

Many genes have DARs at two developmental stages. Compared with the ring stage parasites subjected to the 37 °C control temperature, there are 573 gain DARs in the trophozoite stage group. The top three GO terms enriched in the gain DARs were host cell plasma membrane, antigenic variation, and adhesion of symbiont to microvasculature. Some specific motifs in gain DARs (DARs (GATGATGAHGA (MEME-1), REV (MA2344.1), ATHB-15 (MA1026.3), etc.) are related to differential developmental stages. However, the exact mechanism linking this variability to gene ontology enrichment, including the roles of specific motifs, remains unclear. Our findings identify genes with temperature-dependent promoter accessibility, which correlates with distinct gene expression patterns [50]. Furthermore, chromatin structures provide valuable insights into host–parasite interactions and the parasite environment. Comparative analysis of chromatin structure snapshots across two developmental stages and under varying temperatures revealed dynamic temperature-responsive reorganization and intricate higher-order architectures throughout the parasite transcriptome. By analyzing the motifs corresponding to DARs, a search query in the public database of transcription factors can yield intergroup motifs and transcription factors corresponding to DARs. The identification of temperature responsive transcription factor motifs (e.g., Snail, CRF3, DEAR3, Gli1, Nrf1, pax-2, REV, RFX7,) provides insights into the regulatory networks underlying thermal adaptation, which may be crucial for parasite survival during transmission to the mosquito vector.

We integrated ATAC-seq and RNA-seq data to investigate the relationship between chromatin accessibility and gene expression changes under low temperature stimuli. Notably, the limited concordance between DARs and DEGs (41 genes) likely reflects post-transcriptional regulatory layers, such as RNA secondary structure or translational control, which are known to modulate gene expression in *P. falciparum* [28]. While we identified numerous DARs and DEGs associated with temperature changed, the number of genes exhibiting direct concordance between chromatin accessibility and expression changes was relatively limited (41 genes). This observation aligns with an emerging view of multilayered gene regulation in *P. falciparum*. Firstly, it is well established that post-transcriptional mechanisms, including mRNA stability and translation, exert significant control over final transcript abundance, potentially decoupling promoter accessibility from steady-state mRNA levels [10, 51]. Secondly, the action of ApiAP2 transcription factors is likely highly combinatorial and context dependent [52, 53]. A change in chromatin accessibility may be a prerequisite for binding, but the transcriptional outcome depends on the specific combination of TFs and co-factors recruited. Furthermore, the potential involvement of distal enhancers, while challenging to annotate precisely, could also contribute to this disconnect. Therefore, the limited DAR-DEG overlap likely reflects the intricate regulatory network in the parasite, where chromatin architecture provides a permissive landscape that is then interpreted by additional layers of specific transcriptional and post-transcriptional control.

Furthermore, our focus on promoter-proximal regions (≤ 3 kb upstream) may miss distal enhancers, while combinatorial binding of transcription factors (e.g., ApiAP2s) could explain divergent chromatin-expression relationships. Our analysis identified differentially accessible regions (DARs) in several genes, with both gain and loss of DARs correlating with upregulation or downregulation of gene expression. These findings suggest complex regulatory mechanisms, including the possible synergistic effects of both activating and repressing DARs, that modulate gene function in response to temperature changes. These genes are of significant importance in our study, and we are currently investigating their roles in greater detail. Our ongoing research aims to explore the functional mechanisms underlying their regulation under low temperature stress. Given their potential involvement in key stress response pathways, we are conducting further analyses to better understand their relationships with changes in chromatin accessibility and gene expression.

### Study implications, limitations, and future directions

Notably, the AT-rich genome of *P. falciparum* (≈80% AT) posed challenges for Tn5 transposase efficiency, potentially biasing open chromatin identification. To address this, we optimized sequencing depth (12.1 million valid fragments/sample) and used MACS3 with adjusted parameters. A limitation of this study is the variable ATAC-seq mapping rates (15–88%) across samples, with lower rates concentrated in trophozoite-stage samples. This variability is primarily driven by two factors: (1) stage-specific biological characteristics of trophozoites (e.g., intense DNA replication, altered chromatin compaction) that may reduce Tn5 transposase access, and (2) residual host cell contamination in trophozoite preparations, which introduces nonspecific DNA. To minimize potential bias, we applied strict quality control steps, including exclusion of organellar reads, trimming of low-quality sequences, removal of PCR duplicates, and retention of only uniquely mapped reads. Notably, even samples with lower mapping rates retained sufficient aligned reads for reliable peak calling, and biological replicates showed high reproducibility, supporting the reliability of our key findings on temperature-responsive chromatin dynamics.

To address the challenges posed by the high AT-content genome and ensure robust identification of accessible regions, we have reanalyzed all ATAC-seq data with matched gDNA controls to subtract unspecific Tn5 tagmentation signal, which enhances the reliability of our identified temperature-induced differentially accessible regions (DARs). To address the challenge posed by the ~80% AT-rich genome of *P. falciparum*, we optimized the sequencing depth and employed matched gDNA controls. Following the consolidation of peaks from two replicated samples within each group, we detected accessible regions covering a minimum of 47.49% of all *P. falciparum* genes. Our findings corroborated previous reports indicating that the majority of accessible regions are located within 3 kb upstream of the ATG, which is consistent with studies conducted in parasites and other organisms [13, 34, 54, 55]. The low interstudy overlap between Ruiz et al. and Toenhake et al. is consistent with inherent variability in ATAC-seq data from different experimental setups, while our higher overlap with both studies (33.8–74.4%) highlights the breadth of our dataset. The conservation of ApiAP2 TF motifs in overlapping peaks [39] and the association of RUF6-related accessible sites with *var* gene expression confirm the functional relevance of our identified regions. The high reproducibility of our biological replicates further supports the reliability of our findings on temperature-responsive chromatin accessibility. Ultimately, our analysis resulted in the high-throughput identification of new regulatory regions associated with temperature stimuli in parasites. These observations imply that differences in chromatin accessibility could play additional roles in the nucleus that affect parasite development and differentiation, especially after exposure to low temperature stimuli.

While ATAC-seq data were derived from two parasite stages, this study did not capture genes expressed in other stages, such as the mosquito stage, liver stage, and sexual stages. Nonetheless, it offers crucial insights into chromatin accessibility regions and structural changes induced by low temperature stimuli. Moreover, our analysis of ATAC-seq data revealed potential regulatory elements responsible for developmental transitions in vivo. It is crucial to acknowledge several limitations of our study that the depth may not fully encompass all transcripts, and larger, longer-term studies are needed to explore chromatin structures at very low expression levels. In summary, although the data in our study were obtained from cultured blood samples of parasites and the low temperature may not represent the real ambient temperature in mosquitoes, this study provides important information on the landscape and dynamic chromatin structure of *Plasmodium*. The identification of many DARs and motifs should highlight the potential regulatory roles of these accessible chromatin regions and the molecular mechanisms of RNA transcript expression regulation by DARs.

Studies have shown that aberrant dynamics in chromatin structure can promote cancer progression [56, 57]. Therefore, employing methods to decipher chromatin structure in both these contexts and our field of parasitology will help reveal core mechanisms of gene expression and possible therapeutic strategies for diseases and parasites. Our research also sheds light on how specific chromatin architectures influence gene expression in various stress environments. This study focuses on asexual stages; future work should extend these approaches to gametocytes and mosquito-stage parasites to characterize temperature adaptation fully. Future in vivo investigations under diverse stress conditions[58–63], such as pathogen infection, nutrient availability, oxidative stress, immune dysfunction, xenobiotic and endobiotic metabolism, and glucolipid metabolism, may provide insights into the chromatin structural features of these regions and their regulatory functions not only within the realm of parasitology, but also in related research across other species.

While this study identifies temperature-responsive DARs and their transcriptional associations, future research should focus on the functional validation of these candidate regulatory elements, for example, using CRISPR/Cas9-based genome editing, to definitively establish their causal role in temperature-responsive gene regulation and to evaluate their potential as targets for intervention.

## Methods

### Parasite culture and temperature treatment

*P. falciparum* clone 3D7 was kindly provided by Professor Lubin Jiang (Shanghai Institute of Immunity and Infection, Chinese Academy of Sciences). The O+ red blood cells were sourced from either affiliated hospital or from volunteer donors. The *P. falciparum* 3D7 strain was grown in human O+ red blood cells at 5% hematocrit under standard laboratory conditions, in accordance with the methods established by Trager and Jensen [64]. The synchronization of cultures at the ring stage was achieved by two treatments with 5% D-sorbitol administered 8 h apart as previously described [65]. The cultures were scaled up to achieve a minimum volume of 200 mL for each specified time and temperature.

At 45 h and 70 h after the initial sorbitol treatment, the cultures (~8% parasitemia in 5% hematocrit) were evenly divided and transferred to two different temperatures: 37 °C (representing the standard in vitro culture condition) and 26 °C (the low temperature induction treatment). The experimental temperature of 26 °C was specifically selected to mimic the critical thermal transition *P. falciparum* undergoes during transmission, from the human host (37 °C) to the mosquito vector, which resides in an ambient temperature range of ~26–28 °C. This temperature shift is a well-recognized environmental signal regulating parasite development and gene expression in the transmission phase, and the use of 26 °C aligns with established methodologies in malaria research for investigating parasite adaptive responses to transmission-associated thermal stress [6, 31, 66]. Following a 3-h incubation period at these temperatures, the cultures were harvested via centrifugation at 2000 rpm for 3 min to collect packed red blood cells. After lysing the RBCs with a 0.05% saponin solution, the parasite pellets were washed twice with Phosphate Buffered Saline (PBS) (pH 7.4). The resulting pellets were then either directly utilized for ATAC-seq library preparation or preserved in 10 mL of TRIzol reagent at −80 °C for future RNA extraction. ATAC-seq and RNA-seq were performed on the same parasite cultures to ensure consistency in experimental conditions. For each temperature and stage, samples were split into aliquots for simultaneous ATAC-seq library preparation and RNA extraction, minimizing biological variation between datasets.

### RNA-seq Illumina library construction

For RNA-seq library preparation, we employed two kits, the NEBNext Ultra Directional RNA Library Prep Kit (NEB) and the VAHTS Stranded mRNA-seq Library Prep Kit for Illumina (Vazyme Biotech Co.), following their respective protocols. Initially, 3 µg of total RNA were used to extract mRNA with the Poly-A mRNA Magnetic Isolation Module. The mRNA was fragmented by heating at 94 °C for 7 min in NEBNext First Strand Synthesis Reaction Buffer. First-strand cDNA was synthesized using random primers and ProtoScript II Reverse Transcriptase, with Actinomycin D and Murine RNase Inhibitor. Second-strand cDNA synthesis was carried out at 16 °C for 1 h using Second Strand Synthesis Enzyme Mix. The resulting double-stranded cDNA was purified with 1.8X Agencourt AMPure XP beads. The cDNA library was prepared via incubation with NEBNext-End Prep Enzyme Mix at 20 °C for 30 min, followed by incubation at 65 °C for 30 min. NEBNext adaptors were ligated to the cDNAs using Blunt/TA Ligase Master Mix at 20 °C for 15 min. Fragments ranging from 150 to 200 bp were then purified with AMPure XP beads. Before PCR amplification, the libraries were treated with 3 µl of NEBNext USER Enzyme at 37 °C for 15 min. PCR was performed with NEBNext Q5 Hot Start HiFi PCR Master Mix, NEBNext Universal PCR Primer for Illumina, and Index (X) Primer, under the following conditions: initial denaturation at 98 °C for 30 s, 12 cycles of denaturation at 98 °C for 10 s and annealing/extension at 65 °C for 75 s, and a final extension at 65 °C for 5 min. After purification with the AMPure XP system, the library quality was evaluated using an Agilent Bioanalyzer 2100. Sequencing was conducted on an Illumina HiSeq 2000 system at Vazyme Biotech Co., Ltd. (Nanjing, China) and GENEWIZ Company (Suzhou, China). Paired-end reads were aligned to the *P. falciparum* 3D7 assembly release-68 from PlasmoDB (https://plasmodb.org/common/downloads/release-68/Pfalciparum3D7/), followed by read count collection and estimation of gene expression levels across various samples.

### Real‑time quantitative PCR

For qPCR analysis, 2 μg of total RNA were subjected to DNase treatment to eliminate genomic DNA contamination prior to cDNA synthesis. Complementary DNA was synthesized via the use of either random primer mixtures or oligo dT primers with the Superscript III Reverse Transcriptase Kit (Life Technologies) following the manufacturer’s protocol. Real-time PCR was conducted with 1 μl of cDNA template, 12.5 μl of SYBR master mix (Applied Biosystems), and gene-specific primers in a CFX96 Connect Real-Time PCR Detection System (Bio-Rad). The PCR conditions included initial denaturation at 95 °C for 3 min, followed by 40 cycles of 5 s at 95 °C, 30 s at 55 °C, and 30 s at 72 °C, followed by melt curve analysis from 65 °C to 95 °C with a 0.5 °C increment for 5 s. Transcriptional levels were normalized relative to the housekeeping gene PF3D7_0717700 (serine–tRNA ligase, putative). Primers specific to transcripts in 37 control and 26 treated samples were designed specifically for this study.

### Illumina ATAC-seq library construction

For ATAC-seq, we utilized the methodology developed by Corces MR et al. (2017) as previously detailed [13, 33], with slight modifications. Initially, the parasite pellets were extensively washed until clean and then lysed in cold lysis buffer (10 mM NaCl, 10 mM Tris–Cl, pH 7.4, 0.1% IGEPAL CA-630, and 3 mM MgCl_2_), followed by a 10-min incubation at 4 °C on a rotating mixer. The lysed nucleic suspensions were subsequently incubated in a transposase reaction mixture (2.5 μL of transposase (Illumina), 25 μL of 2X TD buffer, and 22.5 μL of nuclease-free water) and purified with AMPure DNA magnetic beads. During this stage, the transposase accesses the nucleus and selectively fragments DNA in open chromatin regions over 30 min at 37 °C. After transposition, library fragments were amplified via 1X NEBnext PCR master mix and 1.25 μM custom Nextera PCR, following methods outlined in a prior investigation. The final ATAC-seq libraries were assessed via a BioAnalyzer high-sensitivity DNA chip 2100 (Agilent) and Qubit for quantification before deep sequencing was performed on the Illumina HiSeq X Ten platform (San Diego, CA, USA) in 150 PE mode.

### ATAC‑Seq data analysis

Raw sequencing data (FASTQ files) were first analyzed with FastQC (v 0.12.1) to assess read quality (Phred scores ≥ 20), adapter contamination, and GC content distribution.

Adapter trimming and low-quality read filtering were performed using Trim Galore! Version 0.6.10 with parameters: –paired –illumina –quality 20 –phred33 -e 0.01 –length 30 (removes Illumina adapters, trims reads with Phred scores < 20. Instructs Cutadapt to use ASCII + 33 quality scores as Phred scores (Sanger/Illumina 1.9 + encoding) for quality trimming, maximum allowed error rate 0.01, and retains reads ≥ 30 bp). The cleaned paired-end reads were aligned to the *Plasmodium falciparum* 3D7 reference genome (PlasmoDB release 68) using Bowtie2 (v2.5.4) in end-to-end very-sensitive mode [67]. The resulting BAM files were sorted, and low-quality alignments (MAPQ < 30) were filtered out using SAMtools (v1.13) [68]. PCR duplicates were marked and removed using Picard Tools (version 3.4.0; http://broadinstitute.github.io/picard/). Reads mapping to the mitochondrial and apicoplast genomes were also excluded using SAMtools. Tracks were visualized in IGV v2.19.7 with biological replicates overlaid. To assess library quality, fragment size distributions were plotted with ATACseqQC (1.34.0) in R, and transcription start site (TSS) enrichment scores were calculated using the compute Matrixand plot Heatmap functions from deepTools2 (v3.5.4) [69], referencing a curated TSS list for *P. falciparum* [70].

Peak calling: Peak calling was performed using MACS3 (v3.0.3) in paired-end mode (-f BAMPE) to account for the paired-end nature of our data [71]. To correct for the AT-rich bias of the *P. falciparum* genome, a matched genomic DNA (gDNA) control library was used as background (-c gDNA.bam). The effective genome size was set to 23,332,839 bp (-g 23,332,839). The –nolambdaparameter was not used as we included a matched gDNA control. The command callpeak was run with the -Boption to generate bedGraph files for visualization. Peaks were called with a *q*-value threshold of 0.05.

Differential accessibility analysis: Differential accessible regions (DARs) were identified using the DiffBind package (v3.20.0) in R. A consensus peakset was generated from all sample peaks. Read counts for each peak in each sample were obtained using dba.count()with the summits = 200 parameter, which recenters peaks around the summit and constrains them to a fixed width of 401 bp to improve count accuracy. Differential analysis was performed using dba.analyze()with the default DESeq2 method (as implemented within DiffBind) under a paired design to account for biological replicates. DARs were defined as those with *P*-value ≤ 0.05 and an absolute log2 fold change (|log2FC|) ≥ 0.5.

### Motif enrichment analysis

To identify transcription factor (TF) binding motifs enriched in DARs, sequences from gain-DARs and loss-DARs (relative to the 37 °C ring stage control) were extracted using bedtools. De novo motif discovery and enrichment analysis against known databases were performed using the MEME-ChIP suite (v5.5.5) [72]. Sequences were analyzed with the following tools: MEME for discovering novel motifs, DREME to find short, core motifs, and Tomtom to compare identified motifs against the JASPAR 2024 non-redundant vertebrate database [73]. The JASPAR database (http://jaspar.genereg.net/), a comprehensive open-access database of transcription factor binding profiles, was used as the reference database for known motif matching. Motifs with a *P*-value < 0.01 were considered significantly enriched.

## Conclusions

Epitranscriptomics, the study of post-transcriptional RNA modifications, represents another regulatory layer that may interplay with chromatin dynamics to fine-tune gene expression under stress conditions. Chromatin structures are integral to the regulation of numerous biological processes through epitranscriptomics modifications. However, the role of chromatin structural changes in the dynamic regulation of the transcriptome during *Plasmodium* parasite development under temperature stimuli remains elusive. By integrating ATAC-seq and RNA-seq methodologies, we probed global chromatin accessibility during the temperature-sensitive stages of asexual replication in *Plasmodium*, revealing the interplay between chromatin dynamics and differential gene expression. Our findings revealed a pronounced increase in DARs at the ring stage, whereas the trophozoite stage presented no DAR under low temperature stimuli. This observation suggests that low temperature selectively modulates chromatin accessibility and transcriptional activation at the ring stage, indicating heightened sensitivity of early developmental stages to temperature fluctuations.

Although our findings are currently descriptive, the identification of these temperature-responsive regulatory regions provides a foundation for further understanding the complex developmental processes of *P. falciparum*. These regions may play crucial roles in the parasite’s adaptation to different temperatures, which is essential for its survival and transmission. Future studies can build on these findings to explore the functional significance of these regions in more detail. Furthermore, our results underscore the pivotal role of open chromatin structures in orchestrating stage-specific gene expression during the blood stage development of *P. falciparum*. This study presents a comprehensive analysis combining ATAC-seq and RNA-seq data under low temperature stimuli, advancing our understanding of the critical nexus between chromatin structure and the intricate biological processes governing *P. falciparum*. The insights derived from the characterization of these chromatin structures contribute to elucidating host–parasite interactions and may hold promise for guiding future investigations into the development of effective vaccines or innovative antimalarial strategies.

## Supplementary Information

Additional file 1: Figure S1: Quality control and comparative analysis of ATAC-Seq data reproducibility and genomic feature overlap. (A) Venn diagrams illustrating the overlap of ATAC-seq peaks between biological replicates for each experimental group. (B) Overlap analysis of identified ATAC-seq peaks with RUF6 and var gene regions. (C) Motif enrichment analysis comparing identified de novo motifs with published transcription factor binding profiles. The four motifs are referenced from previously reported chromatin accessibility studies (Ruiz et al. 2018 and Toenhake et al. 2018). This figure provides comprehensive quality control metrics and validation analyses, confirming the high reproducibility of biological replicates, the enrichment of peaks in functionally significant genomic regions, and the conservation of regulatory motifs, thereby supporting the reliability and biological relevance of the ATAC-seq dataset generated in this study.

Additional file 2.

Additional file 3.

Additional file 4.

Additional file 5.

Additional file 6.

Additional file 7.

Additional file 8.

Additional file 9.

Additional file 10.

Additional file 11.

Additional file 12.

Additional file 13.

## Data Availability

The ATAC-seq and RNA-seq data used in this study have been deposited in the NCBI Sequence Read Archive (https://www.ncbi.nlm.nih.gov/) under accession number PRJNA645985. The custom code and pipelines used for bioinformatics analysis are available in the following GitHub repository: https://github.com/sdauqyw/ATACSeq.

## Abbreviations

FAIRE: Formaldehyde-assisted isolation of regulatory elements
MAINE: MNase-assisted isolation of nucleosome-bound DNA
ATAC-seq: Assay for Transposase-Accessible Chromatin with high throughput sequencing
DEGs: Differentially expressed genes
DAR: Differentially accessible region

## References

[1] World Health Organization. World malaria report 2024. Geneva: WHO Press; 2024.

[2] Choi L, Pryce J, Garner P. Indoor residual spraying for preventing malaria in communities using insecticide-treated nets. Cochrane Database Syst Rev. 2019;5:CD012688. 10.1002/14651858.CD012688.pub2. 31120132 10.1002/14651858.CD012688.pub2 PMC6532761

[3] Capela R, Moreira R, Lopes F. An overview of drug resistance in protozoal diseases. Int J Mol Sci. 2019;20:5748. 10.3390/ijms20225748. 31731801 10.3390/ijms20225748 PMC6888673

[4] Cao J, Jiang L, Miller LH. Decoding infection and transmission: deciphering the mystery of infectious diseases from data-based research. Decod Infect Transm. 2023;1:1–12. 10.1016/j.dcit.2023.03.001.

[5] Bai Y, Caussinus E, Leo S, Bosshardt F, Myachina F, Rot G, et al. A cis-regulatory element promoting increased transcription at low temperature in cultured ectothermic *Drosophila* cells. BMC Genomics. 2021;22:771. 10.1186/s12864-021-08057-4. 34711176 10.1186/s12864-021-08057-4 PMC8555087

[6] Fang J, McCutchan TF. Thermoregulation in a parasite’s life cycle. Nature. 2002;418:742. 10.1038/418742a. 12181557 10.1038/418742a

[7] Qi Y, Zhu F, Eastman RT, Fu Y, Zilversmit M, Pattaradilokrat S, et al. Regulation of *Plasmodium yoelii* oocyst development by strain- and stage-specific small-subunit rRNA. MBio. 2015;6:1–12. 10.1128/mBio.00117-15. 10.1128/mBio.00117-15 PMC4453563 25759501

[8] Chen X, Shen Y, Draper W, Buenrostro JD, Litzenburger U, Cho SW, et al. ATAC-see reveals the accessible genome by transposase-mediated imaging and sequencing. Nat Methods. 2016;13:1013–20. 10.1038/nmeth.4031. 27749837 10.1038/nmeth.4031 PMC5509561

[9] Ponts N, Harris EY, Prudhomme J, Wick I, Eckhardt-Ludka C, Hicks GR, et al. Nucleosome landscape and control of transcription in the human malaria parasite. Genome Res. 2010;20:228–38. 10.1101/gr.101063.109. 20054063 10.1101/gr.101063.109 PMC2813478

[10] Toenhake CG, Fraschka SA, Vijayabaskar MS, Westhead DR, van Heeringen SJ, Bartfai R. Chromatin accessibility-based characterization of the gene regulatory network underlying *Plasmodium falciparum* blood-stage development. Cell Host Microbe. 2018;23:557–69. 10.1016/j.chom.2018.03.007. 29649445 10.1016/j.chom.2018.03.007 PMC5899830

[11] Wu J, Huang B, Chen H, Yin Q, Liu Y, Xiang Y, et al. The landscape of accessible chromatin in mammalian preimplantation embryos. Nature. 2016;534:652–7. 10.1038/nature18606. 27309802 10.1038/nature18606

[12] Rivera CM, Ren B. Mapping human epigenomes. Cell. 2013;155:39–55. 10.1016/j.cell.2013.09.011. 24074860 10.1016/j.cell.2013.09.011 PMC3838898

[13] Buenrostro JD, Giresi PG, Zaba LC, Chang HY, Greenleaf WJ. Transposition of native chromatin for fast and sensitive epigenomic profiling of open chromatin, DNA-binding proteins and nucleosome position. Nat Methods. 2013;10:1213–8. 10.1038/nmeth.2688. 24097267 10.1038/nmeth.2688 PMC3959825

[14] Mawla AM, van der Meulen T, Huising MO. Chromatin accessibility differences between alpha, beta, and delta cells identifies common and cell type-specific enhancers. BMC Genomics. 2023;24:202. 10.1186/s12864-023-09293-6. 37069576 10.1186/s12864-023-09293-6 PMC10108528

[15] Wu X, Yang Y, Zhong C, Guo Y, Wei T, Li S, et al. Integration of ATAC-seq and RNA-seq unravels chromatin accessibility during sex reversal in Orange-Spotted Grouper (*Epinephelus coioides*). Int J Mol Sci. 2020;21:2800. 10.3390/ijms21082800. 32316525 10.3390/ijms21082800 PMC7215633

[16] Chen D, Chen J, Dai R, Zheng X, Han Y, Chen Y, et al. Integration analysis of ATAC-seq and RNA-seq provides insight into fatty acid biosynthesis in *Schizochytrium limacinum* under nitrogen limitation stress. BMC Genomics. 2024;25:141. 10.1186/s12864-024-10043-5. 38311722 10.1186/s12864-024-10043-5 PMC10840233

[17] Lu Z, Hofmeister BT, Vollmers C, DuBois RM, Schmitz RJ. Combining ATAC-seq with nuclei sorting for discovery of cis-regulatory regions in plant genomes. Nucleic Acids Res. 2017;45:e41. 10.1093/nar/gkw1179. 27903897 10.1093/nar/gkw1179 PMC5389718

[18] Daugherty AC, Yeo RW, Buenrostro JD, Greenleaf WJ, Kundaje A, Brunet A. Chromatin accessibility dynamics reveal novel functional enhancers in *C. elegans*. Genome Res. 2017;27:2096–107. 10.1101/gr.226233.117. 29141961 10.1101/gr.226233.117 PMC5741055

[19] Kaufman CK, Mosimann C, Fan ZP, Yang S, Thomas AJ, Ablain J, et al. A zebrafish melanoma model reveals emergence of neural crest identity during melanoma initiation. Science. 2016;351:aad2197. 10.1126/science.aad2197. 26823433 10.1126/science.aad2197 PMC4868069

[20] Zhao Z, Guo D, Wei Y, Li J, Jia X, Niu Y, et al. Integrative ATAC-seq and RNA-seq analysis of the Longissimus dorsi muscle of Gannan Yak and Jeryak. Int J Mol Sci. 2024;25:6029. 38892214 10.3390/ijms25116029 PMC11172533

[21] Ruiz JL, Ranford-Cartwright LC, Gomez-Diaz E. The regulatory genome of the malaria vector *Anopheles gambiae*: integrating chromatin accessibility and gene expression. NAR Genom Bioinform. 2021;3:lqaa113. 10.1093/nargab/lqaa113. 33987532 10.1093/nargab/lqaa113 PMC8092447

[22] Ding M, Huang W, Liu G, Zhai B, Yan H, Zhang Y. Integration of ATAC-Seq and RNA-Seq reveals FOSL2 drives human liver progenitor-like cell aging by regulating inflammatory factors. BMC Genomics. 2023;24:260. 10.1186/s12864-023-09349-7. 37173651 10.1186/s12864-023-09349-7 PMC10182660

[23] Bevilacqua PC, Ritchey LE, Su Z, Assmann SM. Genome-wide analysis of RNA secondary structure. Annu Rev Genet. 2016;50:235–66. 10.1146/annurev-genet-120215-035034. 27648642 10.1146/annurev-genet-120215-035034

[24] Govindaraju G, Jabeena C, sethumadavan D, Raja N, Rajavelu A. Divulging the enigma of epitranscriptome in *Plasmodium falciparum*. FASEB J. 2019;33:621.3-623. 10.1096/fasebj.2019.33.1_supplement.621.3.

[25] Vandivier LE, Anderson SJ, Foley SW, Gregory BD. The conservation and function of RNA secondary structure in plants. Annu Rev Plant Biol. 2016;67:463–88. 10.1146/annurev-arplant-043015-111754. 26865341 10.1146/annurev-arplant-043015-111754 PMC5125251

[26] Shaw WR, Marcenac P, Catteruccia F. *Plasmodium* development in *Anopheles*: a tale of shared resources. Trends Parasitol. 2022;38:124–35. 10.1016/j.pt.2021.08.009. 34548252 10.1016/j.pt.2021.08.009 PMC8758519

[27] Keim AI, Posfai D, Haystead TA, Derbyshire ER. Heat shock protein 90 is critical for *Plasmodium* parasite liver stage development. FASEB J. 2017;31:604.1-611. 10.1096/fasebj.31.1_supplement.604.1.

[28] Carter R, Miller LH. Evidence for environmental modulation of gametocytogenesis in *Plasmodium falciparum* in continuous culture. Bull World Health Organ. 1979;57:37–52. 397008 PMC2395706

[29] Rawat M, Srivastava A, Johri S, Gupta I, Karmodiya K. Single-cell RNA sequencing reveals cellular heterogeneity and stage transition under temperature stress in synchronized *Plasmodium falciparum* cells. Microbiol Spectr. 2021;9:e0000821. 10.1128/Spectrum.00008-21. 34232098 10.1128/spectrum.00008-21 PMC8552519

[30] Dearsly AL, Sinden RE, Self IA. Sexual development in malarial parasites: gametocyte production, fertility and infectivity to the mosquito vector. Parasitology. 1990;100:359–68. 10.1017/s0031182000078628. 2194152 10.1017/s0031182000078628

[31] Qi Y, Zhang Y, Mu Q, Zheng G, Zhang M, Chen B, et al. RNA secondary structurome revealed distinct thermoregulation in *Plasmodium falciparum*. Front Cell Dev Biol. 2022;9:766532. 10.3389/fcell.2021.766532. 35059397 10.3389/fcell.2021.766532 PMC8763798

[32] Lopez-Barragan MJ, Lemieux J, Quinones M, Williamson KC, Molina-Cruz A, Cui K, et al. Directional gene expression and antisense transcripts in sexual and asexual stages of *Plasmodium falciparum*. BMC Genomics. 2011;12:587. 10.1186/1471-2164-12-587. 22129310 10.1186/1471-2164-12-587 PMC3266614

[33] Corces MR, Trevino AE, Hamilton EG, Greenside PG, Sinnott-Armstrong NA, Vesuna S, et al. An improved ATAC-seq protocol reduces background and enables interrogation of frozen tissues. Nat Methods. 2017;14:959–62. 10.1038/nmeth.4396. 28846090 10.1038/nmeth.4396 PMC5623106

[34] Ruiz JL, Tena JJ, Bancells C, Cortes A, Gomez-Skarmeta JL, Gomez-Diaz E. Characterization of the accessible genome in the human malaria parasite *Plasmodium falciparum*. Nucleic Acids Res. 2018;46:9414–31. 10.1093/nar/gky643. 30016465 10.1093/nar/gky643 PMC6182165

[35] Russell K, Emes R, Horrocks P. Triaging informative cis-regulatory elements for the combinatorial control of temporal gene expression during *Plasmodium falciparum* intraerythrocytic development. Parasit Vectors. 2015;8:81. 10.1186/s13071-015-0701-0. 25652008 10.1186/s13071-015-0701-0 PMC4322800

[36] van Noort V, Huynen MA. Combinatorial gene regulation in *Plasmodium falciparum*. Trends Genet. 2006;22:73–8. 10.1016/j.tig.2005.12.002. 16380193 10.1016/j.tig.2005.12.002

[37] Oladejo D, Oduselu G, Dokunmu T, Isewon I, Okafor E, Iweala EEJ, et al. In silico evaluation of inhibitors of *Plasmodium falciparum* AP2-I transcription factor. FASEB J. 2022;36:L7455. 10.1096/fasebj.2022.36.S1.L7455.

[38] Fan Y, Shen S, Wei G, Tang J, Zhao Y, Wang F, et al. Rrp6 regulates heterochromatic gene silencing via ncRNA RUF6 decay in malaria parasites. mBio. 2020;11:e01110-20. 10.1128/mBio.01110-20. 32487761 10.1128/mBio.01110-20 PMC7267889

[39] Bonnell Victoria A, Zhang Y, Brown Alan S, Horton J, Josling Gabrielle A, Chiu T-P, et al. DNA sequence and chromatin differentiate sequence-specific transcription factor binding in the human malaria parasite *Plasmodium falciparum*. Nucleic Acids Res. 2024;52:10161–10179. 10.1093/nar/gkae585. 38966997 10.1093/nar/gkae585 PMC11417369

[40] Kumar S, Valansi C, Haile MT, Li X, Flyak K, Dwivedy A, et al. Malaria parasites utilize two essential plasma membrane fusogens for gamete fertilization. Cell Mol Life Sci. 2022;79:549. 10.1007/s00018-022-04583-w. 36241929 10.1007/s00018-022-04583-w PMC9568910

[41] Angrisano F, Sala KA, Da DF, Liu Y, Pei J, Grishin NV, et al. Targeting the conserved fusion loop of HAP2 inhibits the transmission of *Plasmodium berghei* and *falciparum*. Cell Rep. 2017;21:2868–78. 10.1016/j.celrep.2017.11.024. 29212032 10.1016/j.celrep.2017.11.024 PMC5732318

[42] Billker O, Shaw MK, Margos G, Sinden RE. The roles of temperature, pH and mosquito factors as triggers of male and female gametogenesis of *Plasmodium berghei in vitro*. Parasitology. 1997;115:1–7. 10.1017/s0031182097008895. 9280891 10.1017/s0031182097008895

[43] Voss TS, Bozdech Z, Bartfai R. Epigenetic memory takes center stage in the survival strategy of malaria parasites. Curr Opin Microbiol. 2014;20:88–95. 10.1016/j.mib.2014.05.007. 24945736 10.1016/j.mib.2014.05.007

[44] Bunnik EM, Venkat A, Shao J, McGovern KE, Batugedara G, Worth D, et al. Comparative 3D genome organization in apicomplexan parasites. Proc Natl Acad Sci U S A. 2019;116:3183–92. 10.1073/pnas.1810815116. 30723152 10.1073/pnas.1810815116 PMC6386730

[45] Tang J, Chisholm SA, Yeoh LM, Gilson PR, Papenfuss AT, Day KP, et al. Histone modifications associated with gene expression and genome accessibility are dynamically enriched at *Plasmodium falciparum* regulatory sequences. Epigenetics Chromatin. 2020;13:50. 10.1186/s13072-020-00365-5. 33225957 10.1186/s13072-020-00365-5 PMC7682024

[46] Toenhake CG, van der Wel A, Wu HY, Kanyal A, Nieuwenhuis IG, van der Werff NM, et al. Epigenetically regulated RNA-binding proteins signify malaria hypnozoite dormancy. Cell Rep. 2023;42:112727. 10.1016/j.celrep.2023.112727. 37392389 10.1016/j.celrep.2023.112727

[47] Yuda M, Iwanaga S, Kaneko I, Kato T. Global transcriptional repression: an initial and essential step for *Plasmodium* sexual development. Proc Natl Acad Sci U S A. 2015;112:12824–9. 10.1073/pnas.1504389112. 26417110 10.1073/pnas.1504389112 PMC4611670

[48] Modrzynska K, Pfander C, Chappell L, Yu L, Suarez C, Dundas K, et al. A knockout screen of ApiAP2 genes reveals networks of interacting transcriptional regulators controlling the *Plasmodium* life cycle. Cell Host Microbe. 2017;21:11–22. 10.1016/j.chom.2016.12.003. 28081440 10.1016/j.chom.2016.12.003 PMC5241200

[49] Sinha A, Hughes KR, Modrzynska KK, Otto TD, Pfander C, Dickens NJ, et al. A cascade of DNA-binding proteins for sexual commitment and development in *Plasmodium*. Nature. 2014;507:253–7. 10.1038/nature12970. 24572359 10.1038/nature12970 PMC4105895

[50] Rovira-Graells N, Gupta AP, Planet E, Crowley VM, Mok S, de Ribas Pouplana L, et al. Transcriptional variation in the malaria parasite *Plasmodium falciparum*. Genome Res. 2012;22:925–38. 10.1101/gr.129692.111. 22415456 10.1101/gr.129692.111 PMC3337437

[51] Bunnik EM, Polishko A, Prudhomme J, Ponts N, Gill SS, Lonardi S, et al. DNA-encoded nucleosome occupancy is associated with transcription levels in the human malaria parasite *Plasmodium falciparum*. BMC Genomics. 2014;15:347:347. 24885191 10.1186/1471-2164-15-347 PMC4035074

[52] Abel S, Le Roch KG. The role of epigenetics and chromatin structure in transcriptional regulation in malaria parasites. Brief Funct Genomics. 2019;18:302–13. 10.1093/bfgp/elz005. 31220857 10.1093/bfgp/elz005 PMC6859822

[53] Watzlowik MT, Das S, Meissner M, Langst G. Peculiarities of *Plasmodium falciparum* gene regulation and chromatin structure. Int J Mol Sci. 2021;22:5168. 10.3390/ijms22105168. 34068393 10.3390/ijms22105168 PMC8153576

[54] Davie K, Jacobs J, Atkins M, Potier D, Christiaens V, Halder G, et al. Discovery of transcription factors and regulatory regions driving *in vivo* tumor development by ATAC-seq and FAIRE-seq open chromatin profiling. PLoS Genet. 2015;11:e1004994. 10.1371/journal.pgen.1004994. 25679813 10.1371/journal.pgen.1004994 PMC4334524

[55] Natarajan A, Yardimci GG, Sheffield NC, Crawford GE, Ohler U. Predicting cell-type-specific gene expression from regions of open chromatin. Genome Res. 2012;22:1711–22. 10.1101/gr.135129.111. 22955983 10.1101/gr.135129.111 PMC3431488

[56] Valencia AM, Kadoch C. Chromatin regulatory mechanisms and therapeutic opportunities in cancer. Nat Cell Biol. 2019;21:152–61. 10.1038/s41556-018-0258-1. 30602726 10.1038/s41556-018-0258-1 PMC6755910

[57] Zhu J, Sammons MA, Donahue G, Dou Z, Vedadi M, Getlik M, et al. Gain-of-function p53 mutants co-opt chromatin pathways to drive cancer growth. Nature. 2015;525:206–11. 10.1038/nature15251. 26331536 10.1038/nature15251 PMC4568559

[58] Li X, Chen L, Liu T, Chen Y, Wang J, Song B. Integrated analysis of ATAC-seq and transcriptomic reveals the ScDof3-ScproC molecular module regulating the cold acclimation capacity of potato. Plant Physiol Biochem. 2024;210:108576. 10.1016/j.plaphy.2024.108576. 38608502 10.1016/j.plaphy.2024.108576

[59] Li M, Li J, Zhang Y, Zhai Y, Chen Y, Lin L, et al. Integrated ATAC-seq and RNA-seq data analysis identifies transcription factors related to rice stripe virus infection in *Oryza sativa*. Mol Plant Pathol. 2024;25:e13446. 10.1111/mpp.13446. 38502176 10.1111/mpp.13446 PMC10950023

[60] He M, Li Y, Li Y, Dong B, Yu H. Dynamics of chromatin opening across larval development in the urochordate ascidian *Ciona savignyi*. Int J Mol Sci. 2024;25:2793. 38474039 10.3390/ijms25052793 PMC10931586

[61] Zhang Y, Jiang M, Xiong Y, Zhang L, Xiong A, Wang J, et al. Integrated analysis of ATAC-seq and RNA-seq unveils the role of ferroptosis in PM2.5-induced asthma exacerbation. Int Immunopharmacol. 2023;125:111209. 10.1016/j.intimp.2023.111209. 37976599 10.1016/j.intimp.2023.111209

[62] Xu F, Cong P, Lu Z, Shi L, Xiong L, Zhao G. Integration of ATAC-Seq and RNA-Seq identifies key genes and pathways involved in the neuroprotection of S-adenosylmethionine against perioperative neurocognitive disorder. Comput Struct Biotechnol J. 2023;21:1942–54. 10.1016/j.csbj.2023.03.001. 36942104 10.1016/j.csbj.2023.03.001 PMC10024148

[63] Qiu F, Zheng Y, Lin Y, Woldegiorgis ST, Xu S, Feng C, et al. Integrated ATAC-Seq and RNA-Seq data analysis to reveal OsbZIP14 function in rice in response to heat stress. Int J Mol Sci. 2023;24:5619. 36982696 10.3390/ijms24065619 PMC10057503

[64] Trager W, Jensen JB. Human malaria parasites in continuous culture. Science. 1976;193:673–5. 10.1126/science.781840. 781840 10.1126/science.781840

[65] Lambros C, Vanderberg JP. Synchronization of *Plasmodium falciparum* erythrocytic stages in culture. J Parasitol. 1979;65:418–20. 383936

[66] Singhaboot Y, Keayarsa S, Piaraksa N, Phumratanaprapin W, Kunawut P, Dondorp A, et al. Temperature dependence of *Plasmodium falciparum* erythrocytic stage development. Am J Trop Med Hyg. 2019;100:1191–5. 10.4269/ajtmh.18-0894. 30938284 10.4269/ajtmh.18-0894 PMC6493921

[67] Langmead B, Salzberg SL. Fast gapped-read alignment with Bowtie 2. Nat Methods. 2012;9:357–9. 10.1038/nmeth.1923. 22388286 10.1038/nmeth.1923 PMC3322381

[68] Li H, Handsaker B, Wysoker A, Fennell T, Ruan J, Homer N, et al. The sequence alignment/map format and SAMtools. Bioinformatics. 2009;25:2078–9. 10.1093/bioinformatics/btp352. 19505943 10.1093/bioinformatics/btp352 PMC2723002

[69] Ramirez F, Ryan DP, Gruning B, Bhardwaj V, Kilpert F, Richter AS, et al. deepTools2: a next generation web server for deep-sequencing data analysis. Nucleic Acids Res. 2016;44:W160–5. 10.1093/nar/gkw257. 27079975 10.1093/nar/gkw257 PMC4987876

[70] Adjalley SH, Chabbert CD, Klaus B, Pelechano V, Steinmetz LM. Landscape and dynamics of transcription initiation in the malaria parasite *Plasmodium falciparum*. Cell Rep. 2016;14:2463–75. 10.1016/j.celrep.2016.02.025. 26947071 10.1016/j.celrep.2016.02.025 PMC4806524

[71] Zhang Y, Liu T, Meyer CA, Eeckhoute J, Johnson DS, Bernstein BE, et al. Model-based analysis of ChIP-Seq (MACS). Genome Biol. 2008;9:R137. 10.1186/gb-2008-9-9-r137. 18798982 10.1186/gb-2008-9-9-r137 PMC2592715

[72] Machanick P, Bailey TL. MEME-ChIP: motif analysis of large DNA datasets. Bioinformatics. 2011;27:1696–7. 10.1093/bioinformatics/btr189. 21486936 10.1093/bioinformatics/btr189 PMC3106185

[73] Fornes O, Castro-Mondragon JA, Khan A, van der Lee R, Zhang X, Richmond PA, et al. JASPAR 2020: update of the open-access database of transcription factor binding profiles. Nucleic Acids Res. 2020;48:D87–92. 10.1093/nar/gkz1001. 31701148 10.1093/nar/gkz1001 PMC7145627

