# Supplementary material for: Integration of ATAC-seq and RNA-seq reveals temperature-responsive regulatory regions in Plasmodium falciparum asexual stages
Source: Parasit Vectors. 2026 Feb 20;19:134. doi: 10.1186/s13071-026-07288-2 (PMC13032594; doi:10.1186/s13071-026-07288-2)
Supplement: Supplementary file 1 — Additional file 1: Figure S1: Quality control and comparative analysis of ATAC-Seq data reproducibility and genomic feature overlap. (A) Venn diagrams illustrating the overlap of ATAC-seq peaks between biological replicates for each experimental group. (B) Overlap analysis of identified ATAC-seq peaks with RUF6 and var gene regions. (C) Motif enrichment analysis comparing identified de novo motifs with published transcription factor binding profiles. The four motifs are referenced from previously reported chromatin accessibility studies (Ruiz et al. 2018 and Toenhake et al. 2018). This figure provides comprehensive quality control metrics and validation analyses, confirming the high reproducibility of biological replicates, the enrichment of peaks in functionally significant genomic regions, and the conservation of regulatory motifs, thereby supporting the reliability and biological relevance of the ATAC-seq dataset generated in this study. [file 13071_2026_7288_MOESM1_ESM.pdf]

A

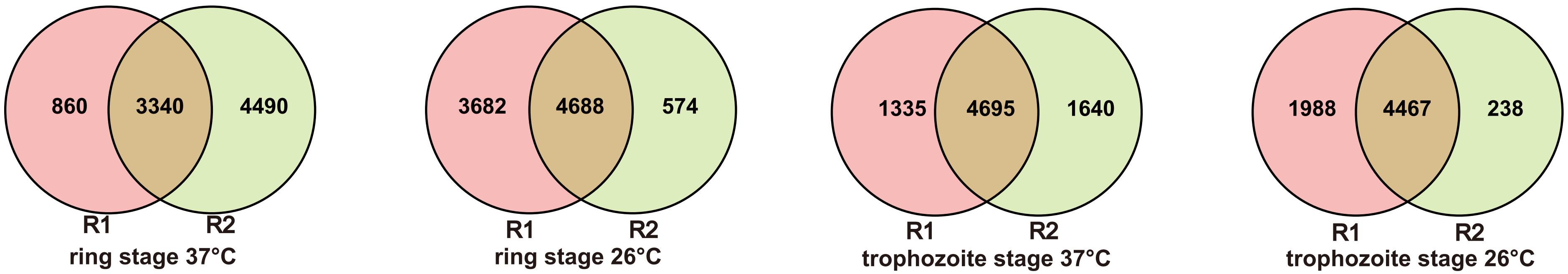

B

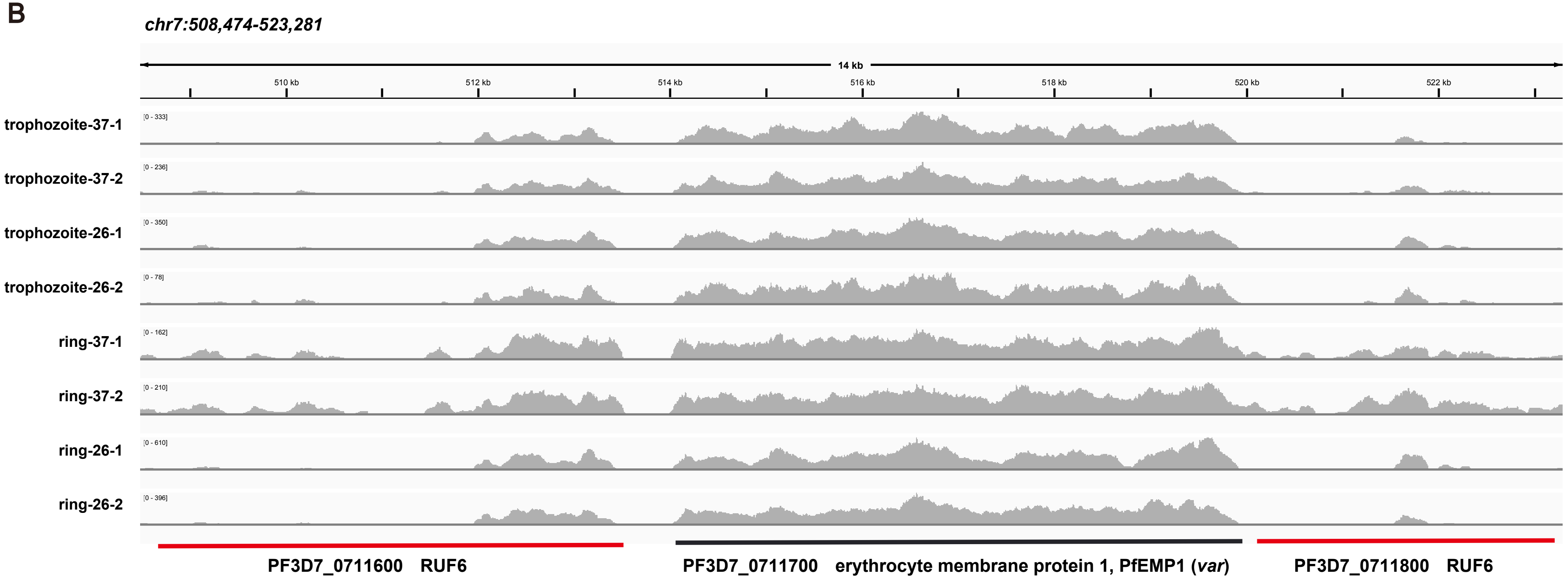

C

motif	sequence	ring_37_1	ring_37_2	ring_26_1	ring_26_2	trophozoite_37_1	trophozoite_37_2	trophozoite_26_1	trophozoite_26_2
AP2-I cognate motif	GTGCA	1482	3121	2765	2686	2383	2131	2643	1985
de_novo_motif_031	TTATTACAC	31	118	61	65	36	59	63	40
de_novo_motif_050	GAGCTCAA	11	37	40	42	29	29	41	35
de_novo_motif_028	GCACTTTTATTGCA	3	7	11	15	3	7	11	15
